# Pathological Changes in Internal Organs after Blocking Low Hydraulic Resistance Channels along the Stomach Meridian in Pigs

**DOI:** 10.1155/2013/935687

**Published:** 2013-06-26

**Authors:** Wen-Ting Zhou, Shu-Yong Jia, Yu-Qing Zhang, Yu-Ying Tian, Guang-Jun Wang, Tao Huang, Li Pang, Yong-Sheng Zhou, Xue-Yan Sun, Wei-Bo Zhang

**Affiliations:** ^1^Institute of Acupuncture & Moxibustion, China Academy of Chinese Medical Sciences, 16 Nanxiaojie, Dongzhimennei, Beijing 100700, China; ^2^World Federation of Chinese Medicine Societies, Beijing 100029, China; ^3^Institute of Chinese Materia Medica, China Academy of Chinese Medical Sciences, Beijing 100700, China

## Abstract

*Objective.* The correlation between meridians and organs (Zang-fu) is an important aspect of meridian theory. The objective of this paper is to investigate the pathological changes in the organs resulting from blocking low hydraulic resistance channel (LHRC) along the stomach meridian by injecting gel in pigs so as to offer some insight into the correlation between meridians and internal organs. *Methods.* Four white piglets and twelve black minipigs were divided into four batches and were observed in different periods. Each batch included two pairs of pigs and each pair matched two pigs with similar conditions among which gel was injected into 6~8 low hydraulic resistance points along the the stomach meridian in the experimental pig and the same amount of saline was injected into the same points in the control pig. The state of stomach and intestine was observed 6~10 weeks after the blocking model was developed. *Results.* The results showed that there were bloated stomach or/and intestine in all the experimental pigs while there were normal states in seven control pigs except one dead during the experiment. *Conclusion.* The findings confirmed that the blockage of LHRC along the stomach meridian can influence the state of stomach and intestine, leading to a distension on stomach or/and intestine.

## 1. Introduction

Meridians and collaterals are critical system in keeping health by transporting Qi-Blood, nourishing tissue, communicating between somata and viscera, and balancing Yin-Yang according to the classic theory of traditional Chinese medicine (TCM) [1]. If a meridian gets a problem, a stasis, for instance, the running of Qi-Blood will be slow or even stopped and the organ related to the meridian may become disordered and gradually develop a disease. This important pathological view in TCM is quite different from the one in Western medicine. It is unbelievable to the actions of the meridian from the view of Western medicine as no meridian structure has been observed using anatomic technique. However, countless clinical effects of acupuncture indicate that there is an unknown regulating system in our body which is related to the meridians and the acupoints on the meridians.

The correlation between meridians and internal organs is an important aspect of meridian theory. Acupuncturists treat a visceral disease through needling the acupoints located on the related meridian. A systematic analysis was made recently on the correlation between acupoints and pathological changes of the internal organs through reviewing of literatures in the database of CNKI (China's National Knowledge Infrastructure) from 1959 to 2011 and the database of Pubmed (US National Library of Medicine) in the past 10 years. The result showed that specificity was found on the pathological changes of Zang-fu (viscera) organs when acupoints were stimulated [2]. The influence of meridians to the organs was confirmed through acupoint stimulation while it remained a secret if there is a pathological change in an organ when the related meridian gets a problem. To answer the question, we must firstly answer what are the meridians and the Qi in meridian channels. In Yellow Emperor's canon of internal medicine, Qi was defined to fill into the physique and moisten the fine hair like the dew moistening the grasses and woods [3]. Qi can also transform into five kinds of fluid: when the weather is cold and one's clothes are thin, most of them will be changed into urine and vapour; when the weather is hot in summer and one's clothes are thick, most of them will be wet; when one's mood is in sorrow and the energy merges into the heart, they will transform into tears; when there is heat in the middle warmer and the stomach energy is flaccid and slow, they will change into saliva. When the evil energy is blocking inside, the Yang energy is obstructed and fails to circulate; edema will occur [4]. The unique thing owning these properties is the interstitial fluid (IF) which occupies about 20% of our body volume. IF exists among many tiny holes constructed by collagen and fine fibers of glycosaminoglycan in extracellular space. The early idea of IF comes from Guyton et al. in 1966 who claimed that IF is gel-like substance which is almost immobile [5]. But this point of view was challenged by many scientists in Europe like Aukland and Reed [6] and Levick [7] who published large review articles to prove the existence of two phase of IF, one is gel, the other is free fluid which can flow in tissue. In 2007, Swartz and Fleury discussed the functions of IF flow on cell-cell signaling and morphogenesis [8].

The flow of IF in porous medium follows Darcy's law (1856) that the flux is positively proportional to pressure gradient and negatively proportional to the hydraulic resistance (HR) of tissue [7]. And the fluid must follow the conservation equation which means it must flow continuously without compression [7]. In 1997, Zhang put forward a hypothesis that meridians are the tissue which has lower HR and IF will flow toward the meridians and along the meridians according to the laws [9]. 

This hypothesis was basically proven by finding a series of low HR points along meridians, a good transportation of IF pressure wave along meridians, and a migration of isotope along meridians in Zhang's lab in 90s in the twentieth century. A low hydraulic resistance channel (LHRC) along meridians was finally discovered which may represent partly the essence of meridians [10]. 

 The further study is to identify the function of LHRC. One of the functions is in pathological aspect. According to Yellow Emperor's canon, meridian channels have a pathological state of blockage which is the main cause of many diseases. To establish a blocking model of meridian channels, Xu et al. developed a method by injecting hydrogel into low HR points along meridians and measured the transportation of IF pressure wave to determine if the channel was really blocked. The pathological model of blockage of meridian (main) channels was successfully developed in 2007 [11] that brought a new starting point in Zhang's research. The upcoming study started from an occasional observation that two minipigs were found to appear gaseous distention in stomach several weeks after the LHRC along the stomach meridian was blocked by gel in the experiments in December 2008 and March 2009, respectively. This phenomenon attracts the authors to observe intently the state of stomach and intestine after the stomach meridian was blocked so as to test the ancient theory of meridian-organ correlation.

## 2. Materials and Methods

### 2.1. Animal Conditions

**Figure 1:**
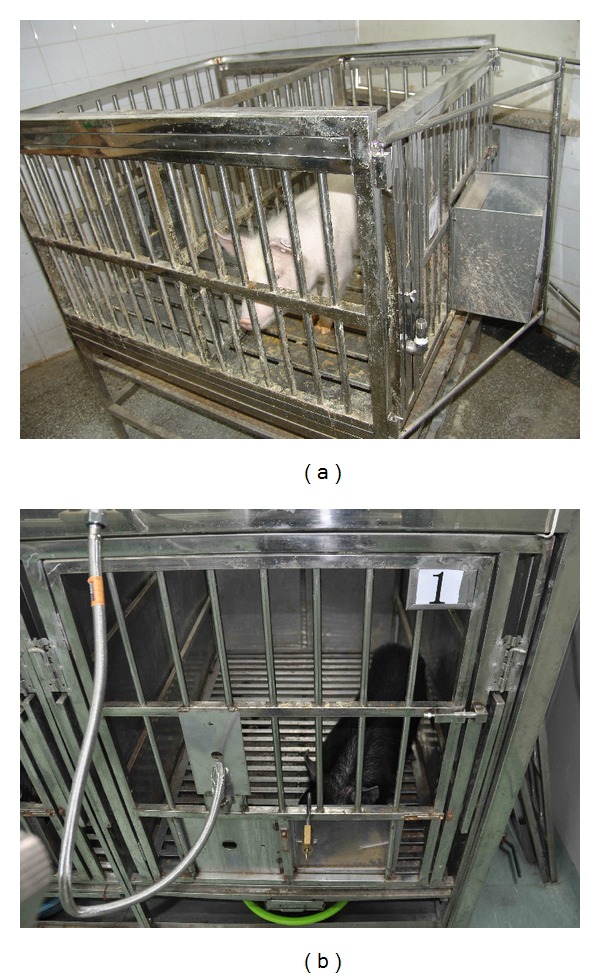
The cages used to house the pigs. (a) Cage used in the first batch of pigs (1.1 × 0.5 × 0.7 m for each pig). (b) Cage used in the other three batches of pigs (1.1 × 0.7 × 0.8 m).

The experiment was carried out on four batches of healthy pigs. The first batch contained four white piglets which were obtained from Beijing veterinary training center (weight: 10~12 kg, male, a month of age). The four pigs were divided into two groups each of which contained one experimental pig with gel injection and one control pig with saline injection. Each pair of pigs was housed simultaneously in a stainless steel cage (1.1 × 1.0 × 0.7 m) separated by two parts using a fence (each part: 1.1 × 0.5 × 0.7 m, Figure 1(a)). The food and water were given three times a day.

The second, third, and fourth batches of experiments were carried on 12 Chinese black minipigs, 4 pigs in each batch, from Beijing Kexing Experimental Animal Breeding Center. The pigs were temporarily housed in the animal laboratory at 20 ± 2°C with a relative humidity of 50 ± 10% (license number: SYXK Beijing 2011-0014) in the Institute of Chinese Materia Medica, China Academy of Chinese Medical Science, for detailed observation. The cages have a size of 1.1 × 0.7 × 0.8 m and separated to two equal parts to house two pigs. The pig was feed twice a day with 150 g pig fed in the morning and 100 g in the evening and had accessed ad libitum to tap water. The light was simulated day and night by turning on the light at 8:00 am and turning off the light at 8:00 pm automatically. The experiment was approved by the Institutional Animal Care and Use Committee (license number: 120101) of Institute of Chinese Materia Medica, China Academy of Chinese Medical Sciences.

### 2.2. Gel Preparation

In the first batch of pigs, “Amazing” polyacrylamide hydrogel was got from Jilin Aodong medical material company in China. One gram of original Polyacrylamide hydrogel was diluted by mixing 5 mL of 0.9% saline which made it possible to be injected. As the gel could not be bought later, a new gel, “Macrolane”, a stable hyaluronic acid from Uppsala company in Sweden, was used in the other pigs from batch 2 to batch 4. The gel has enough liquidity and can be injected directly into tissue without dilution. To see if the gel was still at the injected position after so many breeding days, a 10 mL gel package from Sweden was mixed by 0.3 mL 1% Alcian Blue (8 GX, Sigma Co., USA) for marking the gel from batch 2 to batch 4.

### 2.3. Experimental Procedure

**Figure 2:**
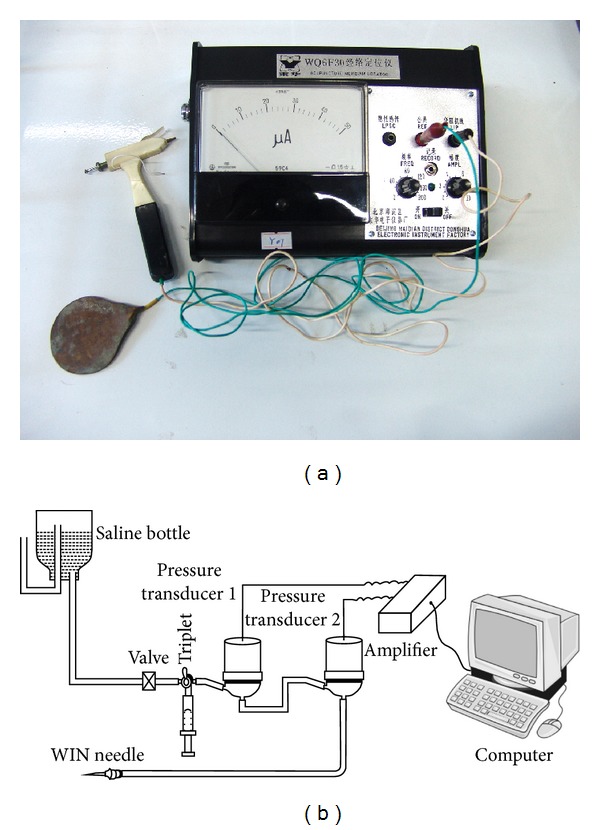
The equipment for measuring the meridians. (a) The WQ6F30 low impedance meridian locator. (b) The pressure differential continuous hydraulic resistance detector.

**Figure 3:**
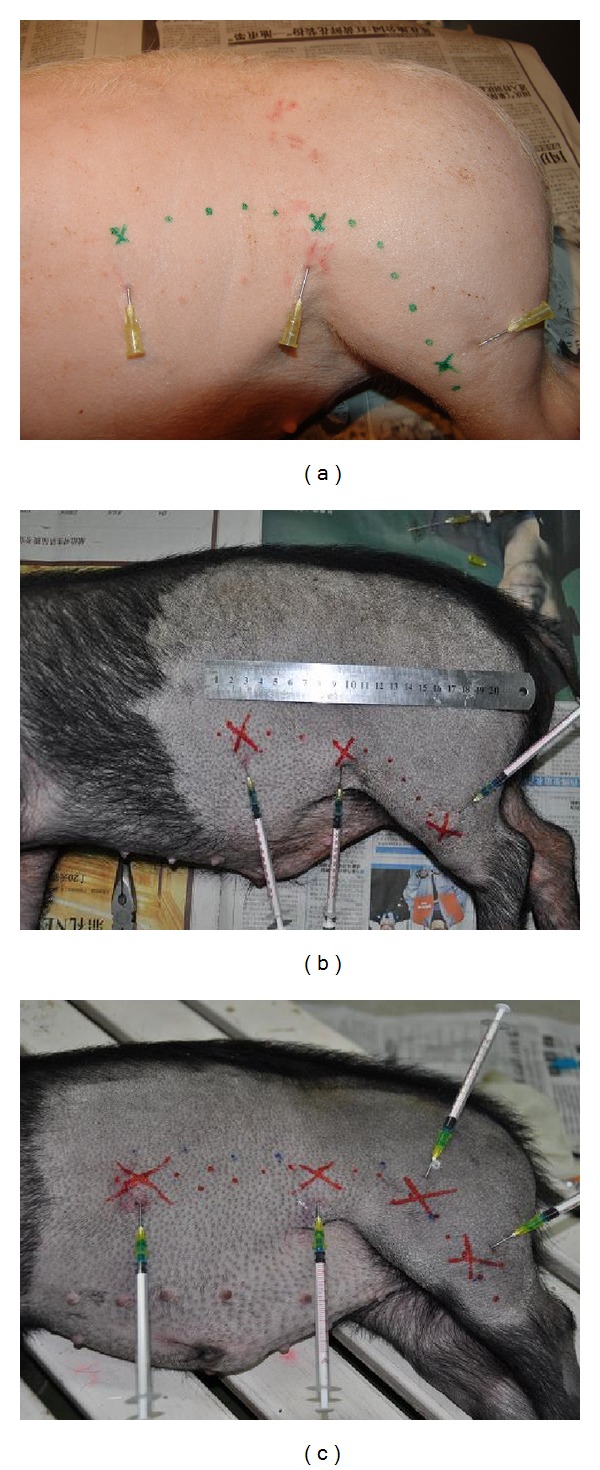
The positions of three injected points along one side of the stomach meridian in the first batch (a), second and third batches (b) and fourth batch (c) of pigs.

The pigs were housed for about one week to adapt to the new environment. Fasting was carried out 12 hours before the operation. Then they were anaesthetized by pentobarbital sodium (30 mg/kg) intraperitoneally in batch 1 (4 piglets) and by injecting phenobarbital sodium solution (0.3 mg/kg) and Xylazine Hydrochloride Injection (0.1 mg/kg) intramuscularly in batches 2, 3, and 4 (12 pigs). The low impedance meridian lines on pigs were firstly measured by a low impedance meridian locator (type: WQ6F30, made in Donghua Company in China, Figure 2(a)) according to the previous study [12]. The LHRC along the stomach meridian was then measured by a pressure differential continuous HR detector (Figure 2(b)) which was described in detail in previous papers [10, 13]. After a low hydraulic resistance point (LHRP) was found in subcutaneous tissue along the stomach meridian, the needle was fixed on skin by glue and small amount of polyacrylamide hydrogel was slowly injected into the LHRP through the measured needle to block the channel. For the batches 1 to 3, totally six points were injected symmetrically on both sides with three on one side along the stomach meridian. One point was on leg close to Housanli (corresponding to Zusanli (ST36) on Human), the other was on hip at groin level, and another was on trunk at the third nipple level (Figures 3(a) and 3(b)). The injected volume on leg was 0.5 mL, 0.7 mL on hip, and 1.0 mL on trunk. In the fourth batch of pigs, the injected points were increased to eight, four at one side among which two points were at calf and thigh. The injected volumes were 0.5 mL, 0.5 mL, 0.7 mL, and 0.9 mL from calf to trunk (Figure 3(c)).

After the channel was blocked, the pig was sent back to the cage to feed for one to two months. The behavior was observed by a 24 hour monitor and the image was stored in computer and could be stored in anytime. Finally the pig was anaesthetized again and the state of stomach and intestine was checked by exploratory laparotomy, watching and taking pictures using a digital camera (Nikon D5000, made in Japan). Then the stomach was cut open with scissors and inner surface was exposed to check if there was a gastric ulcer or perforation. The gel which was injected into the meridian channels was also checked during the final operation by uncovering the skin.

## 3. Results

### 3.1. The States of Stomach and Intestine

**Figure 4:**
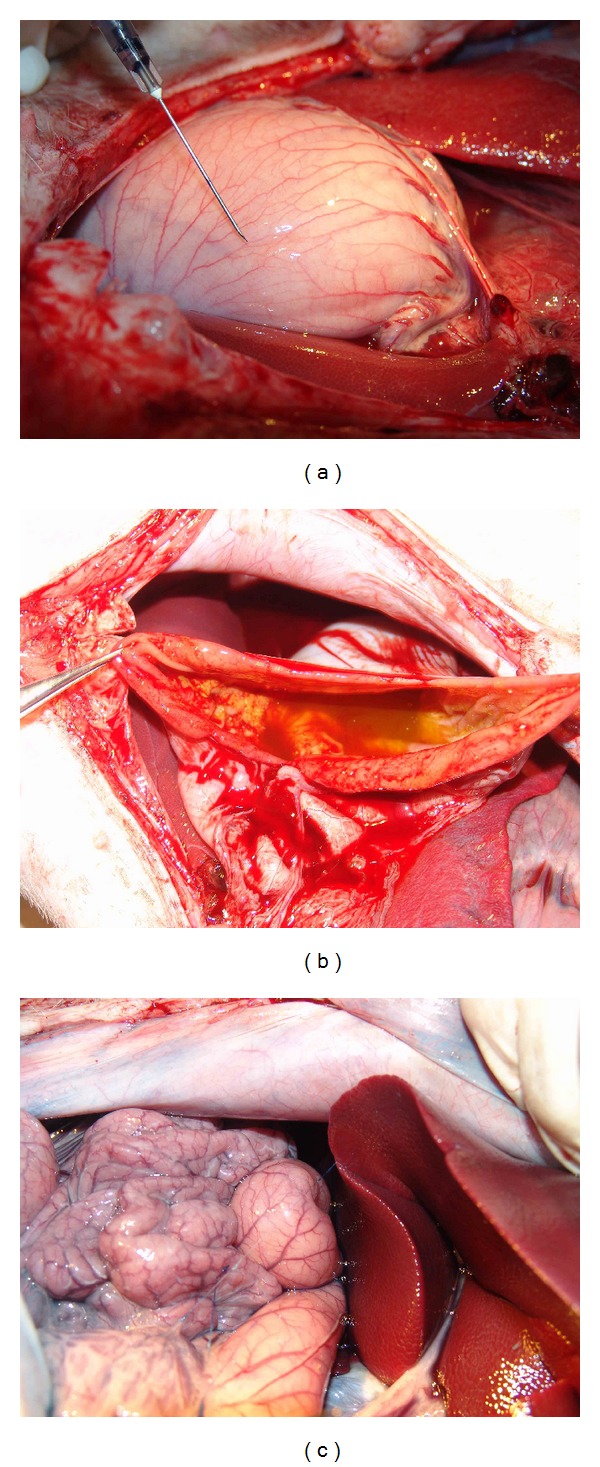
(a) A bloated stomach was observed on the third pig. (b) Only small amount of liquid was found in the stomach. (c) The stomach is normal in the control pig by saline injection.

In the first pair of pigs among the first batch one month later, the pig whose stomach meridian was blocked by gel injection exhibited stomach distension (Figure 4(a)) where only small amount of liquid was found inside the stomach after opening it (Figure 4(b)) while the stomach is normal in the control pig (Figure 4(c)).

**Figure 5:**
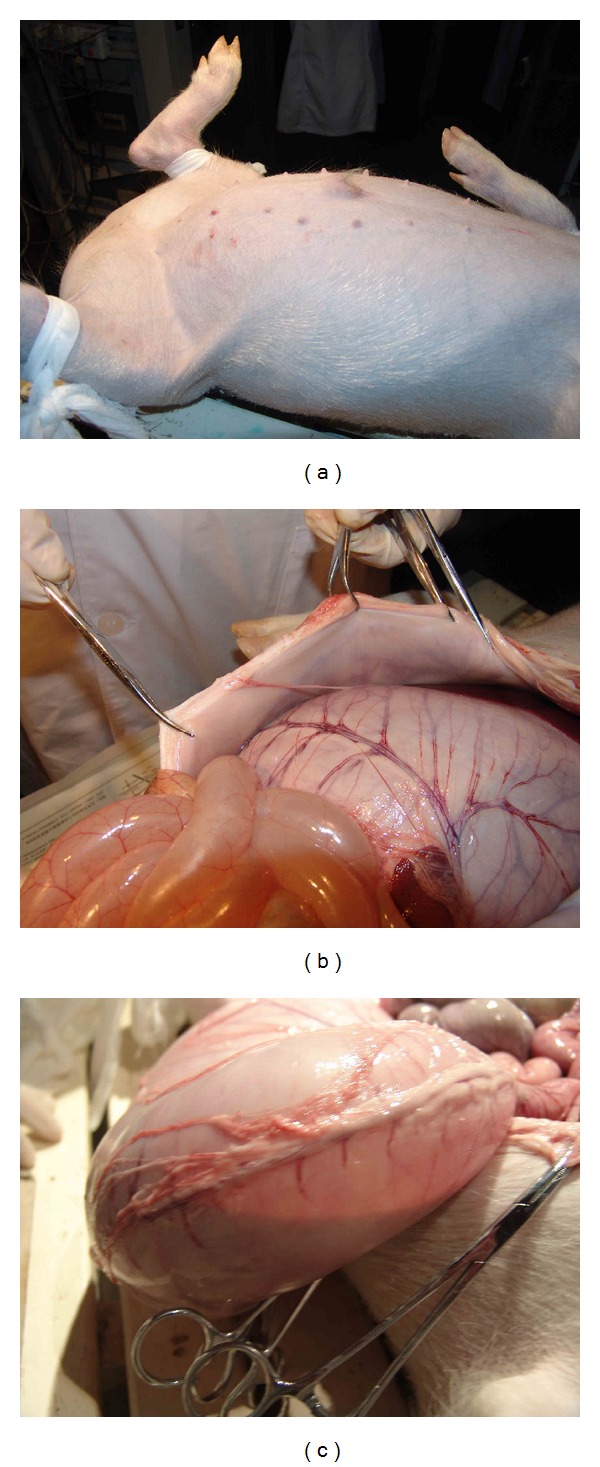
The pig exhibited bloated abdomen (a) and bloated stomach and intestine (b). (c) The stomach was filled with gas and water.

**Figure 6:**
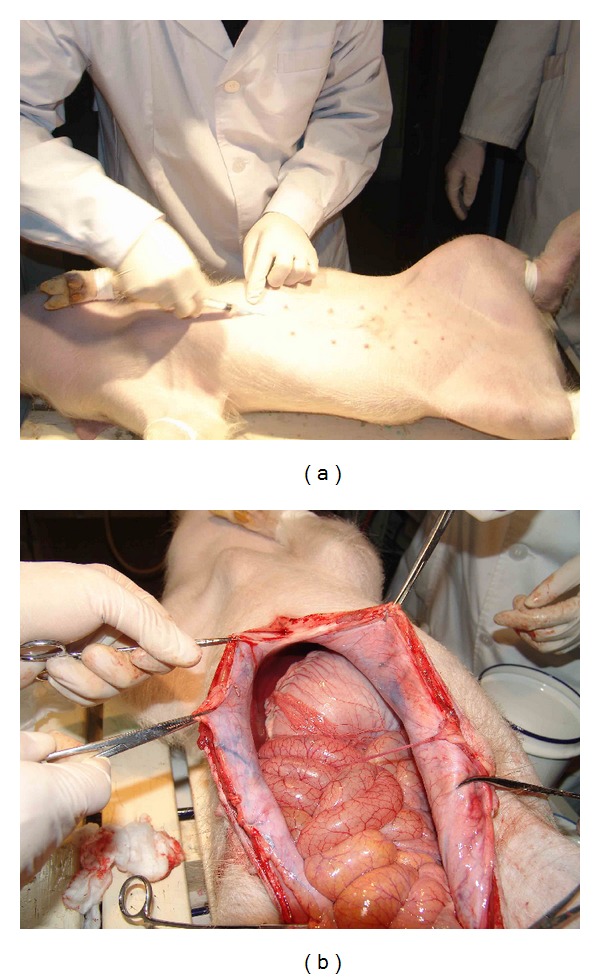
The control pig exhibited normal abdomen (a) and normal stomach and intestine (b).

For the other pair of pigs among the batch 1 after one and a half months, the pig which was injected with gel exhibited serious abdominal distention (Figure 5(a)) and meteorism not only in stomach but small intestine as well (Figure 5(b)). The stomach was filled with about 2/3 gas and 1/3 liquid when watched in light (Figure 5(c)). The control pig which was injected with saline had a normal abdomen (Figure 6(a)), stomach, and intestine (Figure 6(b)).

**Figure 7:**
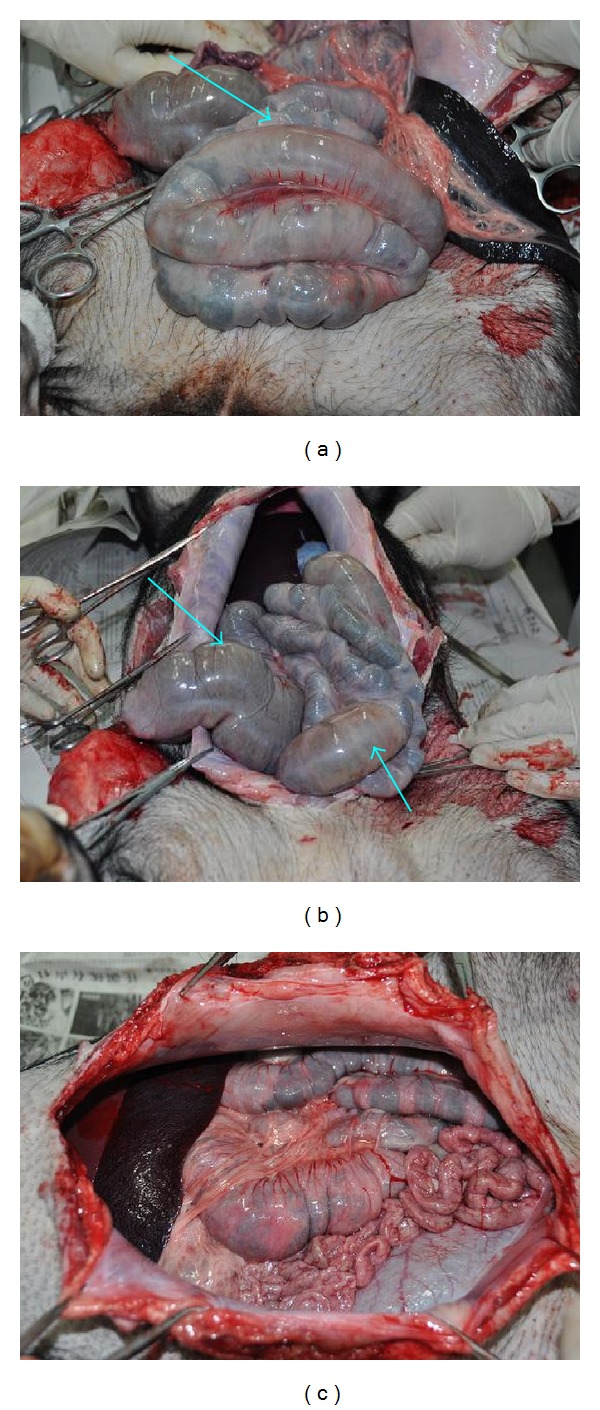
The large intestine at transverse colon (a) and ileocecum (b) exhibited bloated state (↑) in the first experimental pig (batch 2). (c) The control pig showed normal state of stomach and intestine.

The second batch of pigs was experimented from March to June in 2012 containing four minipigs which were divided two pairs with similar weights in each pair, containing an experimental pig and a control pig. The result showed obvious bloated parts at transverse colon (Figure 7(a)) and ileocecum (Figure 7(b)) but the stomach was normal in the first experimental pig after 72 days of gel injection while it was normal on both stomach and intestine in the control pig (Figure 7(c)) in a similar duration of 70 days after the operation.

**Figure 8:**
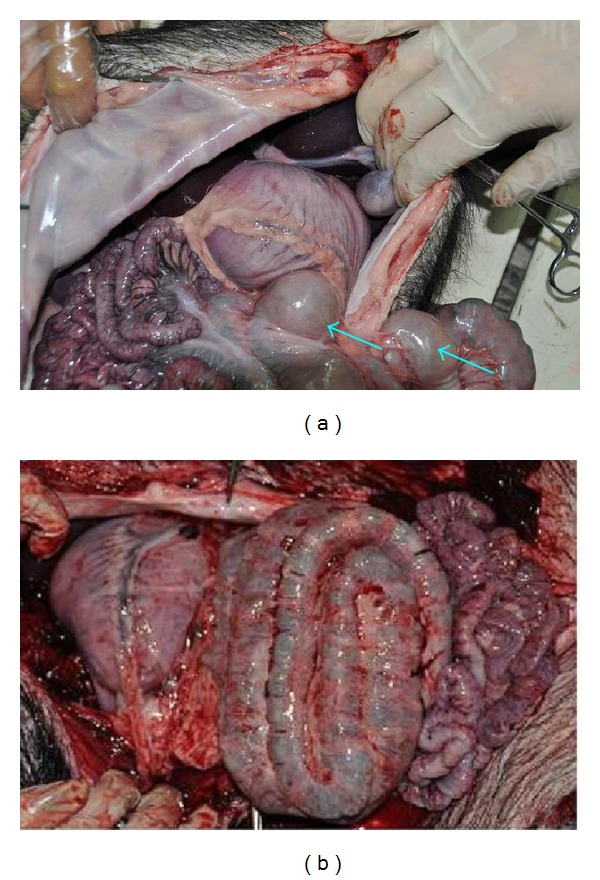
(a) In the experimental pig of second pair, there were two parts (↑) on large intestine appear bloated state. (b) There was normal state on stomach and intestine in the control pig.

For the experimental pig in the second pair, the stomach and small intestine were normal while two bloated parts were found on large intestine after 59 days of the injection (Figure 8(a)). The control pig had a normal stomach and intestine after the same duration (Figure 8(b)).

**Figure 9:**
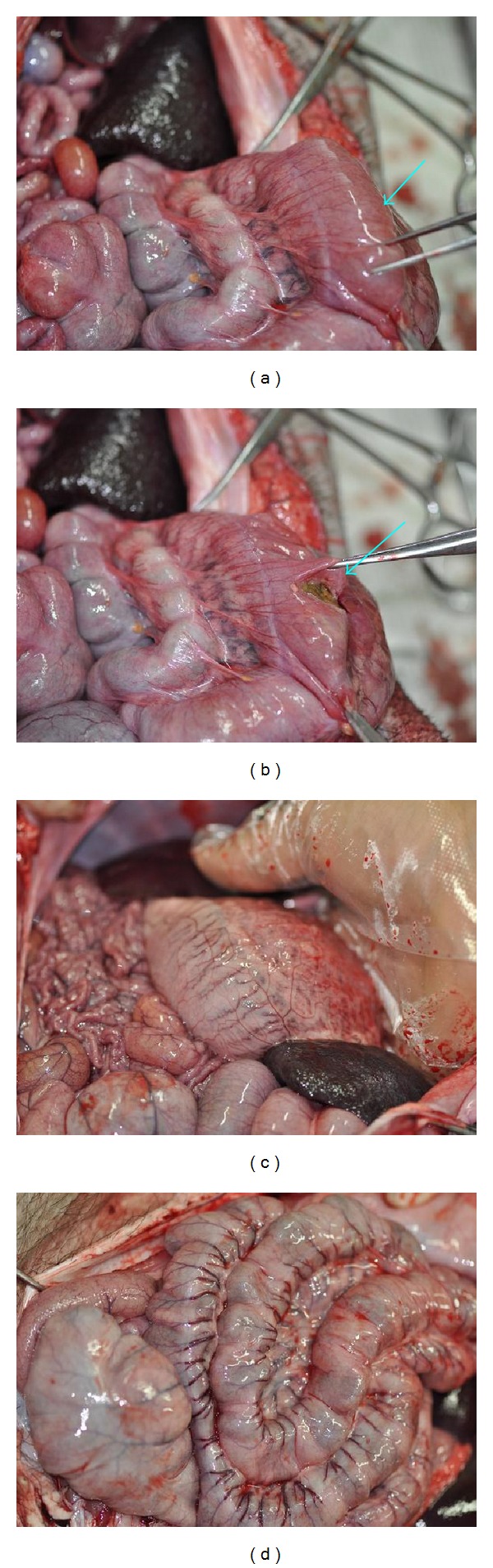
In the experimental pig of the first pair (batch 3), there was a bloated part on large intestine (a) and it shrank when cutting it open with scissors (b). The control pig showed normal state of stomach (c) and intestine (d).

The third batch of pigs was exprimented from August to September of 2012. In the first pair of pigs lasted for 52 days experimental pig and control pigs lasted for 55 days between the injecting date and operating date. The result showed a distension at one part of large intestine (Figure 9(a)). The bloated part obviously shrank when cutting it open with scissors (Figure 9(b)), indicating the existence of gas in it. The stomach and small intestine were normal in the control pig (Figures 9(c) and 9(d)).

**Figure 10:**
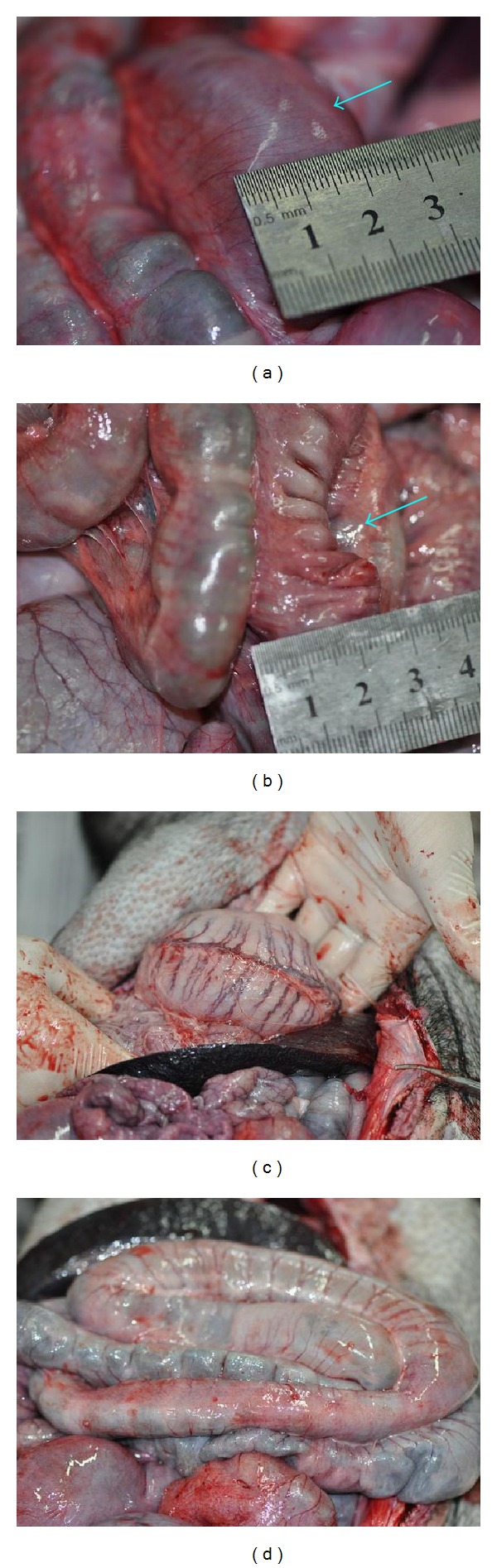
In the experimental pig of second pair (batch 3), there was a flatulence on large intestine (a) and there was strikingly shrink after releasing the gas in it (b). There was normal state in stomach (c) and intestine (d) in the control pig.

In the second pair of pigs, the experimental pig exhibited similar situation to that of the experimental pig in the first pair. There was a striking shrink after releasing the gas by cutting it open (Figures 10(a) and 10(b)) while the state of stomach and intestine was normal in the control pig (Figures 10(c) and 10(d)).

**Figure 11:**
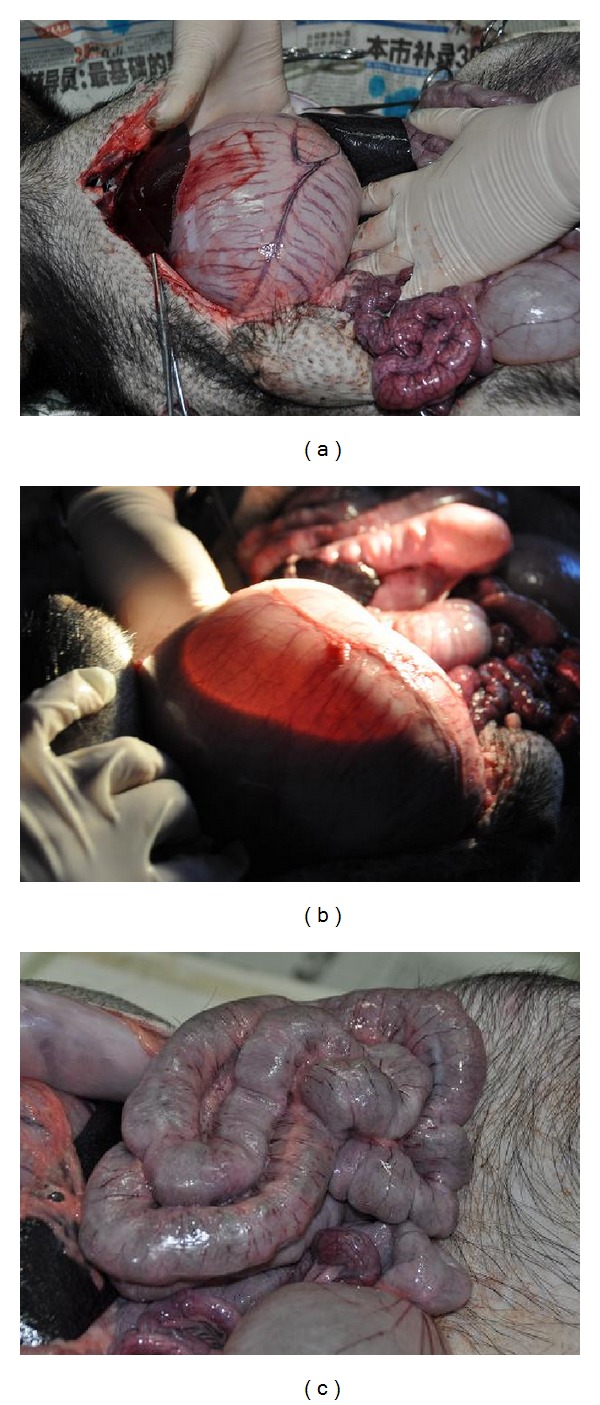
In the experimental pig of the first pair (batch 4), there was a bloated stomach (a). Gas and water were observed in the stomach (b). There was a normal state in stomach and intestine (c) in the control pig.

In the fourth batch of pigs was experimented from December 2012 to February 2013 eight points along the stomach meridian were blocked. The first pair of pigs lasted for 45 days experimental pig and control pig lasted for 47 days between the injecting date and operating date. After opening the abdominal cavity, it was found that the stomach was enlarged strikingly (Figure 11(a)). By lighting it, gas and liquid could be seen in the stomach (Figure 11(b)) which was similar to the situation in the first batch of experimental pig (Figure 5(c)). There was a normal state of stomach and intestine in the control pig (Figure 11(c)).

**Figure 12:**
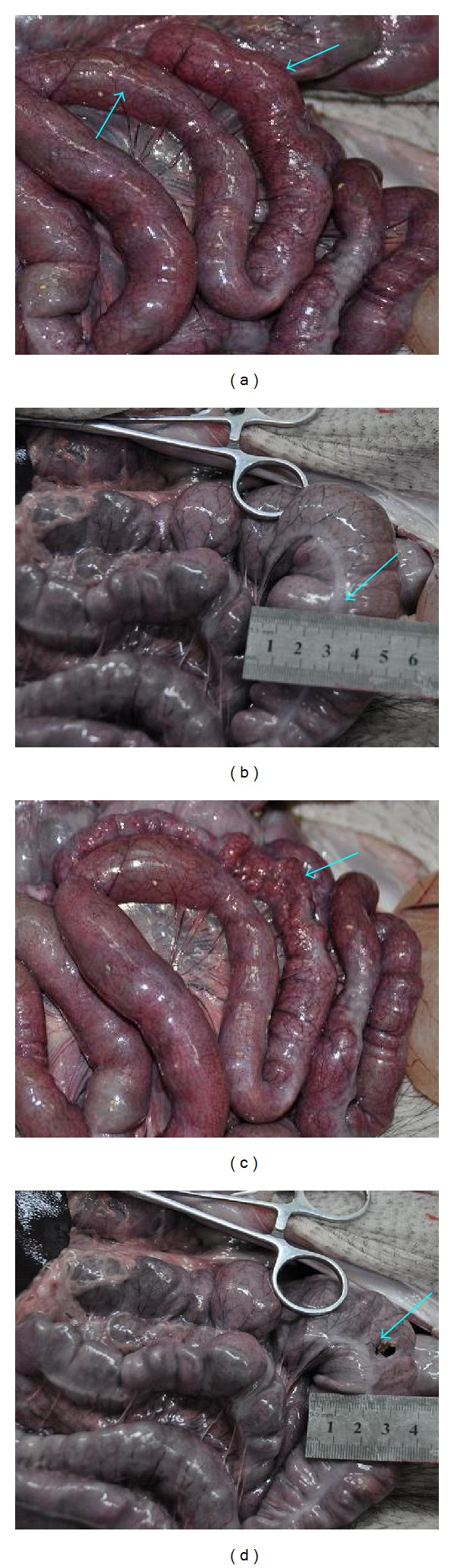
In the experimental pig of the second pair (batch 4), there were bloated parts (↑) on small intestine (a) and large intestine (b). The intestine shrank strikingly (↑) after the inner gas was released by cutting a small hole with scissors ((c), (d)).

In the second pair of pigs, the experimental pig exhibited distensions both on small intestine (Figure 12(a)) and large intestine (Figure 12(b)). The size immediately shrank strikingly (Figure 12(c)) after the gas in small intestine was released by cutting open a hole with scissors. A quantitative measurement was made to show a size from 5 cm in diameter to 3.5 cm after releasing the gas in large intestine (Figure 12(d)). However, the stomach have not appeared obvious abnormal. The control pig in this pair was dead on the second day after the injection.

### 3.2. The Examination of Inner Surface of Stomach

**Figure 13:**
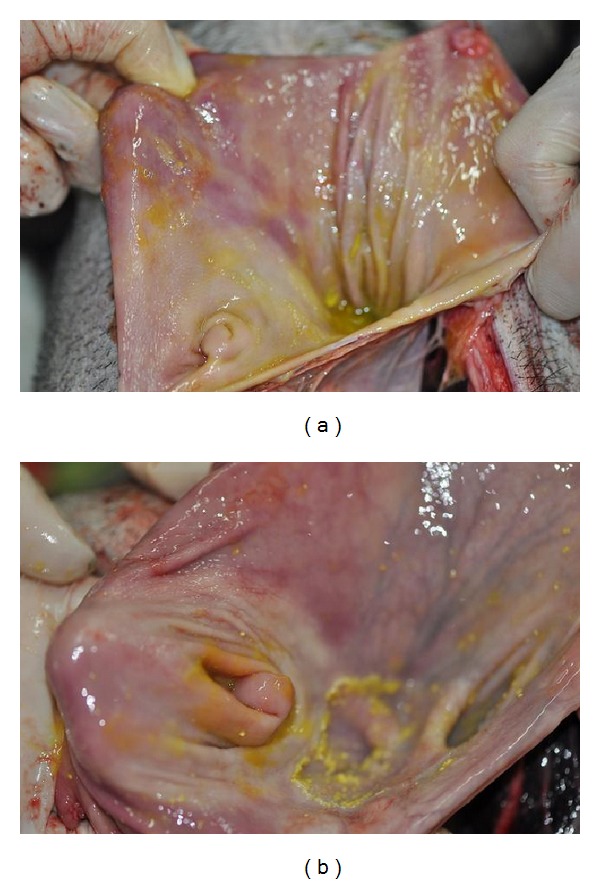
The inner surface of stomach which showed no gastric ulcer or perforation both in an experimental pig (a) and a control pig (b).

The inner surfaces of stomach were examined after the operation to see if there was a gastric ulcer or perforation. No gastric ulcer or perforation was found in all the pigs no matter what was injected into the meridian. The inner surface of stomach was smooth and fresh. The inner surfaces of stomach in a pair of pigs were shown in Figures 13(a) and 13(b).

### 3.3. The Locations of Injected Gel

**Figure 14:**
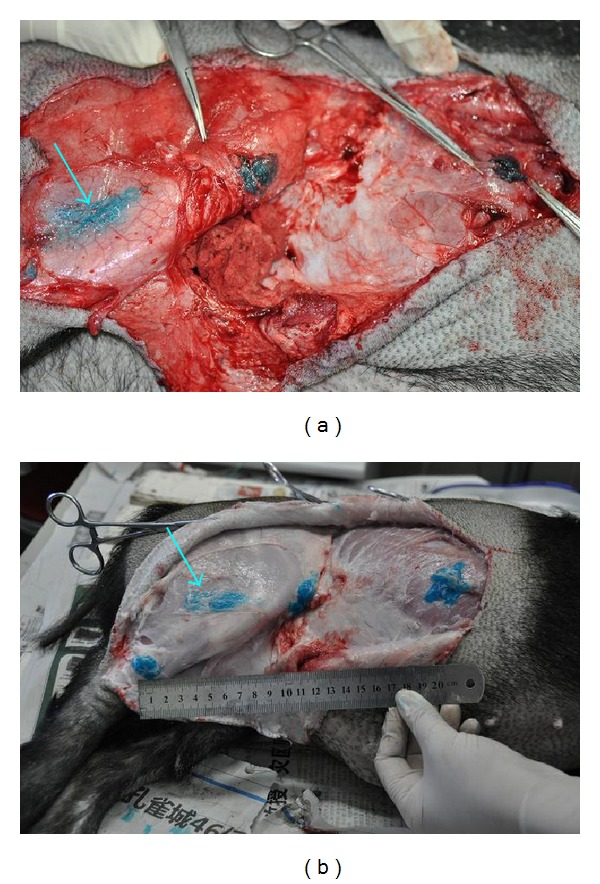
The locations of injected gel along the stomach meridian. (a) Three points were blocked on one side of pigs. (b) Four points were blocked on one side of pigs. The gel at thigh diffused along the stomach meridian (↑).

 The locations of injected gel were checked by uncovering the skin around the injecting points. The gel with blue color could be found in every injected points. The gel on thigh above Zousanli (ST36) diffused a short distance (2–5 cm) along the stomach meridian in all the six pigs while the gel stayed at the injected points or just a slight diffusion (3 cases) at the points on trunk (Figure 14(a)). In the fourth batch of pigs four points along one side of stomach meridian were blocked; the gel on calf can stay at the point without diffusion (Figure 14(b)).

## 4. Discussion

 The results showed a coincident pathological change in stomach or intestine after LHRC along the stomach meridian was blocked.

### 4.1. The Stomach Meridian and the Related Internal Organs

Most studies on correlation between meridians and internal organs were carried out through observing the organ response when stimulating a meridian point or observing the changes along a meridian when the pathological model of a related organ was developed. This is the first time to study the pathological chronic influence of meridian on the related organs which has obvious importance to understand the deep mechanism of diseases and the principle of health. 

Stomach meridian is one of the longest meridians in our body which relates not only stomach but large intestine and small intestine according to the chapter of Ben Shu in Yellow Emperor's canon of internal medicine. Both upper sea point of large intestine Shangjuxi (ST37) and lower sea point of small intestine Xiajuxi (ST39) locate on stomach meridian. So an overall observation of stomach, small intestine, and large intestine was taken in the blocking model of the stomach meridian. 

### 4.2. The Observation of Pathological State of Organs

Necropsy is a common method to test the pathological state of organs. The size or volume is often used to examine the organ state in veterinary pathological anatomy [14]. But it is difficult to get quantitative measurements as the shape of organs is very irregular. We tried to measure the diameter of intestine by a ruler but found it very difficult. So the judgment was made by eyes in the most cases and clear photos were collected by CCD camera as much as possible.

An abnormal distension of stomach or intestine was defined when large amount of gas exists inside. It is usually determined not only by the size or volume but the color, transparency, curvature, and elasticity. A real distension of intestine with a lot of gas often exhibits yellow or grey color with transparent wall which is soft and elastic. We developed a method to test the contained gas by cutting a hole to release the gas and taking pictures again in the later experiments. The usual situation in pathological stomach is abnormally much liquid and gas in the stomach. We also developed a simple method by lighting the stomach to show the volume of gas and liquid inside the stomach. As the observation took a long time and the conditions and materials were improved gradually, it is hard to keep constant conditions in all the pigs during the whole study. However, we kept each observation containing one pig with gel injection and the other pig with saline injection with the same conditions so as to let them comparable.

The results showed that all the 8 pigs, no matter white or black, had a problem on stomach or/and intestine with a bloated state where there were mostly gas and/or liquid (in stomach) after injecting gel into the stomach meridian while the states of stomach and intestine were basically normal in 7 control pigs in which the same amount of saline was injected at the same points. The result illustrated that the exhibited pathological state in pigs was not caused by the operation and the injection itself but the content of injection. No gastric ulcer or perforation was found in all the pigs which implied that the pathological change is mainly functional during the observed period of two months. But we could not get a conclusion that the blockage of meridian can only cause a functional influence on the internal organ. After an enough long time, a functional pathological change may develop an organic change which needs to be verified in later experiments.

### 4.3. The Technique of Blocking Meridian

The technique of blockage is often used in biology to see if a pathway or signal really exists to cause an effect. A transient blockage of meridian by mechanical pressure has also been used in meridian study for a long time in Hu XL's team in Fujian province. The results showed a break of acupuncture effects when a mechanical pressure was applied on related meridian line [15, 16]. But to observe a long-term influence of meridian blockage, mechanical pressure is not convenient. The main component of hydrogel is hyaluronic acid which was found to have a very high hydraulic resistance by Scott et al. [17] and Coleman et al. [18]. When the gel was injected into the tissue, it will be filled into the tiny pores of the tissue, resisting the flow of interstitial fluid along LHRC and breaking the transmission of chemical signals along the channel. This blocking effect is similar to the mechanical pressure which can shrink the space of gaps in the interstitium while the gel can block the channel for a long time as long as it stays in the tissue. However, as LHRC is a porous medium channel which is opened to any directions, it is hard to be totally blocked like blocking a blood vessel. The interstitial fluid can still flow round the injected gel and continue to move while the flux will be weaken. In the phenomenon of propagated sensation along meridian (PSM), the sensation induced by acupuncture or acupress can move around a scar on the meridian [19] which illustrates the porous medium characteristics of the channel. Therefore three or four points were blocked on each side of stomach meridian to enhance the blocking effect. As the blocking effect will be accumulated following the time, an average blocking time of 49 days was estimated from previous experience that most pigs have not exhibited a bloated state of stomach or intestine after stomach meridian was blocked by gel in a period from two to four weeks and the earliest pig was found to have the problem 41 days after the blockage. But to verify the relationship quantitatively between the days of undergoing blocking model and the pathological degree, more pigs should be observed.

The located situation of gel in meridian channel is also a factor to influence the pathological effect. In the early experiment of two white pigs (batch 1) and even earlier two black pigs which were bred in a smaller cage, the main pathological changes happened on stomach while it rarely appeared in the later pigs which lived in a bigger cage. They had more space to move their limb that might cause a diffusion of gel at thigh (Figure 13) and diminish the blocking effect. The diffusion on thigh level was found on the second and third batches of pigs. After this was noticed, we added a new injecting point at calf level on the fourth batch of pigs and found that the gel could stay there without a diffusion; at the same time there were a distension on stomach in the first experimental pig and a distension on small intestine in the second experimental pig which had not been found in batch 2 and batch 3. So it was impressive that the blocking degree and allocation can influence the bloated position in viscera.

### 4.4. The Mechanism of Interaction between Meridians and Organs

How does a meridian influence an internal organ? The observed gastrointestinal flatulence in pigs is in coincidence with “abdominal distension” in the symptoms of stomach meridian. In the view of Western medicine, gastrointestinal flatulence is a common problem on human which is induced mainly by the abnormal movement of stomach and intestine. The movements are influenced by food, humoral signals such as enterogastrone, and neural signals from enteric nervous system or central nerve system. Earlier study showed a somatic projection from Zusanli to nucleus solitaries which control the movement of stomach and intestine [20]. Zhou et al. found, when giving an electric acupuncture at Zusanli (ST36), Tianshu (ST25), and Liangmen (ST21) on the stomach meridian, the migrating motor complex (MMC) was enhanced [21]. It seems that there is an interaction between meridian and nerve system. As an extracellular fluid channel, how does the channel influence the activity of nerve? In 1986, a new hypothesis was put forward by Agnati et al. [22] that the interactions between neurons are not only through the neural fibers and synapse but also through extracellular space which was named volume transmission (VT). Several review articles concerning VT were published recently [23, 24], indicating the hypothesis has been accepted. Fuxe further pointed out that VT may also happen in peripheral tissue via interstitial fluid transmission [25] which relates to Zhang's discovery of LHRC [10]. The PSM phenomenon which tightly relates to meridians can be understood through VT along LHRC [26]. The idea and the new findings in this work imply that LHCR is the essence of meridians at least in part. Assuming the activity of stomach and intestine was controlled by a series of autonomic nerves and these nerves can interact through LHRC along meridians, whenever LHRC is blocked, the interaction becomes weak, leading to a difficulty of harmonious motion along stomach and intestine. Therefore an accumulation of gas happens gradually in stomach or intestine. 

This is just a preliminary observation to show the existence of long-term pathological influence on internal organs from peripheral meridians. More controlled experiments should be done to see if the stomach meridian is specific to cause the pathological state on stomach and intestine.

## 5. Conclusion

The blockage of LHRC along the stomach meridian can influence the state of stomach and intestine, leading to a pathological distension on stomach or/and intestine. But we could not get a conclusion that the blockage of meridian channel can only cause a functional distention without organic changes as the observing time is still short.
